# Supplementary material for: Characterization of Pathway-Specific Regulator NigR for High Yield Production of Nigericin in Streptomyces malaysiensis F913
Source: Antibiotics (Basel). 2022 Jul 13;11(7):938. doi: 10.3390/antibiotics11070938 (PMC9312159; doi:10.3390/antibiotics11070938)
Supplement: Supplementary file 1 [file antibiotics-11-00938-s001.zip › antibiotics-1792362-supplementary.pdf]

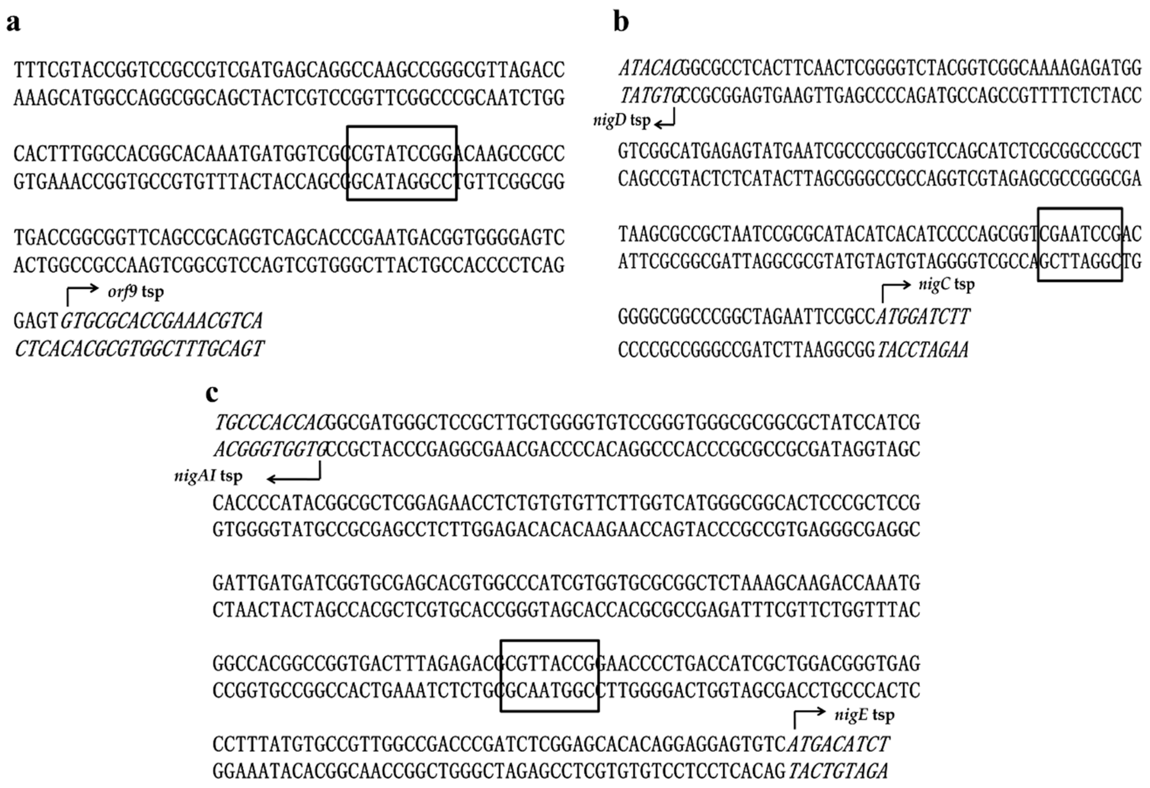

Figure S1. Nucleotide sequence and consensus sequence in the promoter regions of target genes. (**a**) promoter region of *orf9*; (**b**) intergenic regions of *nigD-C*; (**c**) intergenic regions of *nigAI-nigE*. The translational start position (tsp) is indicated by a bent arrow. The consensus sequences are marked by boxes. The coding regions of target genes are italicized.

Table S1. Deduced functions of genes in the nigericin biosynthetic gene cluster

Usage	Proposed Function in Nigericin Biosynthesis	Identity and Similarity (%) with Corresponding Protein in *Streptomyces* sp. DSM4137
orf9	Function unknown	99, 99
nigAVII	Polyketide synthase	97, 97
nigAVIII	Polyketide synthase	98, 98
nigAIX	Polyketide synthase	98, 99
nigBI	Cyclase	99, 100
nigBII	Cyclase	99, 100
nigCI	Epoxidase	99, 99
nigAX	Polyketide synthase	98, 98
nigAXI	Polyketide synthase	99, 100
nigD	Cytochrome P450	99, 99
nigCII	Thioesterase	99, 99
nigAVI	Polyketide synthase	97, 97
nigAV	Polyketide synthase	97, 97
nigAIV	Polyketide synthase	97, 97
nigAIII	Polyketide synthase	98, 98
nigAII	Polyketide synthase	97, 97
nigAI	Polyketide synthase	98, 98
nigE	O-methyltransferase	99, 99
nigR	SARP family Regulator	99, 100
orfL1	Prolyl-dipeptidyl aminopeptidase	99, 99

Table S2. Strains and plasmid used in this work

Strain	Characteristics	Source
Escherichia coli		
DH5α	Host for all plasmids cloning procedures	Invitrogen
ET12567 (pUZ8002)	Donor strain for *E. coli* - *Streptomyces* conjugation	[39]
Streptomyces malaysiensis		
F913	Wild-type producer for nigericin	[22]
DM01R	*nigR* mutant, construct with *nigR* replaced by *aac(3)IV*, Apr^R	This work
DM01Rc	DM01R complementation stran, with a *nigR* under the control of its own promoter, Apr^R	This work
F913-hrdBR	F913 carrying pSET152::P_{hrdB}R	This work
F913-kasOR	F913 carrying pSET152::P_{kaso}R	This work
F913-pSET152	F913 carrying pSET152	This work
Plasmid		
pUC119::*neo*	pUC119 carrying *neo*	[11]
pSET152	Apr^R, integrative *E. coli*-*Streptomyces* shuttle vector	[40]
pSET152sv::*nigR*::*neoR*	Suicide vector containing the *aac(3)IV* and *nigR*-flanking sequences	This work
pSET152::P_{hrdB}R	pSET152 containing *nigR* under the control of *hrdB* promoter	This work
pSET152::P_{kaso}R	pSET152 containing *nigR* under the control of *kasO** promoter	This work

Table S3. Primers used in this work

Name	Sequence (5′-3′)	Usage
NigRdm-upF	AATTAAGCTTTACTCGTCCCAGATGGCGCCG	Construction of pSET152sv::*nigR*::*neoR*
NigRdm-upR	AATTTCTAGAGATCGGCGCGGACGAGAAGAA	Construction of pSET152sv::*nigR*::*neoR*
kanF	AATTTCTAGATACGCCAAGCTTGCATGCCTGC	Construction of pSET152sv::*nigR*::*neoR*
kanR	AATTTCTAGAGAGCTCGGTACCCGAACCCCAG	Construction of pSET152sv::*nigR*::*neoR*
NigRdm-dnDF	AATTTCTAGAGGATGACCGGCTGGAGCTGCA	Construction of pSET152sv::*nigR*::*neoR*
NigRdm-dnDR	GGCCGGTGAGCCCTTCCTCAG	Construction of pSET152sv::*nigR*::*neoR*
nigRNP-F	AATTTCTAGACTCCTCATCAAAGCGGCAACAA	Construction of pSET152::nigRc
nigRNP-R	AATTGAATTCCTGCCGTCGGACACATAACTGATG	Construction of pSET152::nigRc
nigR-CDSF	AATCATATGGGGCACATCGTGCGATATGAG	Construction of pSET152::P$_{hrdB}$R and pSET152::P$_{kasO}$R
nigR-CDSR	AATGAATTCTCAGGCGGGCACATGCAGCTCCAG	Construction of pSET152::P$_{hrdB}$R and pSET152::P$_{kasO}$R
PhrdBF	AATTTCTAGACCCGATCTCCGGCTGTGACC	Construction of pSET152::P$_{hrd}$B
PhrdBR	ATTCATATGGAACAACCTCTCGGAACGTTGGAAA	Construction of pSET152::P$_{hrdB}$R
PkasOF	CTAGATGTTCACATTCGAACGGTCTCTGCTTTGACAACATGCTGTGCGGTGT TGTAAAGTCGTGGCCAGGAGAATACGACAGCGTGCAGGACTGGGGGAGTT	Construction of pSET152::P$_{kasO}$R
PkasOR	TATGAACTCCCCCAGTCCTGCACGCTGTCGTATTCTCCTGGCCACGACTTTA CAACACCGCACAGCATGTTGTCAAAGCAGAGACCGTTCGAATGTGAACA	Construction of pSET152::P$_{kasO}$R
RT-orf9F	GTGGGTGTGGCAGCCGTGAG	RT-PCR analysis of *orf9*
RT- orf9R	CTTCACGCCGGTTGTCTGCC	RT-PCR analysis of *orf9*
RT-AVIIF	GGAACACGCGACGGTCCAAG	RT-PCR analysis of *nigAVII*

RT-AVIIR	TGGTGGTGGGCAGATCGATG	RT-PCR analysis of *nigAVII*
RT-AVIIIF	GAGGCGGTCGATGTGCAGAT	RT-PCR analysis of *nigAVIII*
RT-AVIIIR	GACCACTCCAGGTGAACGGG	RT-PCR analysis of *nigAVIII*
RT-AIXF	CGTCCTATGGGCCGTCATGA	RT-PCR analysis of *nigAIX*
RT-AIXR	CGCCTCCTCGATGCTCTCCT	RT-PCR analysis of *nigAIX*
RT-BIF	CCGCGGATGTCGATGGACTT	RT-PCR analysis of *nigBI*
RT-BIR	AGCAATGTCCGACCTCCCCC	RT-PCR analysis of *nigBI*
RF-BIIF	CGATGAGGCGACCCTCAAGA	RT-PCR analysis of *nigBII*
RF-BIIR	TTACATCGGACTGGGGCTGC	RT-PCR analysis of *nigBII*
RT-CIF	ACTGGCTGTCGGCGCTTGAT	RT-PCR analysis of *nigCI*
RT-CIR	CGCTGGAGCTCGGTGTCCAG	RT-PCR analysis of *nigCI*
RF-AXF	GACACCGCGTGTTCGTCGTC	RT-PCR analysis of *nigAX*
RF-AXR	CGAGTCGGCCGGTCGATATG	RT-PCR analysis of *nigAX*
RT-AXIF	TCAAGGAGACGGATCCGCGA	RT-PCR analysis of *nigAXI*
RT-AXIR	ACGCCTCGGCGAGATAGTGG	RT-PCR analysis of *nigAXI*
RT-DF	GCCCGGAAGATTGTCGACGA	RT-PCR analysis of *nigD*
RT-DR	GGCGACGACGAGCAGGATGA	RT-PCR analysis of *nigD*
RT-CIIF	GTGGTGGCCGGTCACTCCTG	RT-PCR analysis of *nigCII*
RT-CIIR	GTCTCCTTGATGCGGGAGCG	RT-PCR analysis of *nigCII*
RT-AVIF	GGACTACGCCTCGCACAGCC	RT-PCR analysis of *nigAVI*
RT-AVIR	GGTGGAGCGTGGTCCAGTCG	RT-PCR analysis of *nigAVI*
RT-AVF	TCACCACGGACCTGGACACC	RT-PCR analysis of *nigAV*
RT-AVR	TTGCAACGCCAACTCCTCGA	RT-PCR analysis of *nigAV*
RT-AIVF	CGTTGGTGCTACCGGAGTCG	RT-PCR analysis of *nigAIV*
RT-AIVR	CGCGTCACCAGGGAGTCAAC	RT-PCR analysis of *nigAIV*

RT-AIIIF	GGCCTCACCCCCTCCTTCCT	RT-PCR analysis of *nigAIII*
RT-AIIIR	TAGCGCGTCACACCTTCGCC	RT-PCR analysis of *nigAIII*
RT-AIIF	CAACTGCTGTCGGGTCTGGG	RT-PCR analysis of *nigAII*
RT-AIIR	CCACACCCACCTGCTCGAAC	RT-PCR analysis of *nigAII*
RT-AIF	GGCGACCGAACTGAGCGAAC	RT-PCR analysis of *nigAI*
RT-AIR	GTCGAATTCGGACGCGTCGT	RT-PCR analysis of *nigAI*
RT-EF	GCTTCTCCAGATGGCGTGGG	RT-PCR analysis of *nigE*
RT-ER	AGGTGACCACACCGGGATCG	RT-PCR analysis of *nigE*
RT-RF	GAGCGAAAGCCCCGTTCTCA	RT-PCR analysis of *nigR*
RT-RF	TCGAGCCCGAGTTCCTCGTT	RT-PCR analysis of *nigR*
RT-L1F	GACGCCACCGGCAACTACAA	RT-PCR analysis of *orfL1*
RT-L1R	CGCCTCGCTCAGCTTGGTGT	RT-PCR analysis of *orfL1*
F913hrdB-F	CGAGGCTGACCAGATTCCGC	RT-PCR analysis of *hrdB*
F913hrdB-R	CTCCTGCTCGGCGTTGAGCA	RT-PCR analysis of *hrdB*
qRT-AVIIF	CGGGACGATGGAACTCGGTCAG	qRT-PCR analysis of *nigVII*
qRT-AVIIR	GCGAGATCGAGGTCCTGGGTGA	qRT-PCR analysis of *nigVII*
qRT-DF	AAGCGGGACTGCGAGGTG	qRT-PCR analysis of *nigD*
qRT-DR	GAACTGATAGGGGTCGGTGAAGA	qRT-PCR analysis of *nigD*
qRT-CIIF	TTCGCTGTCCCGGCGCATGT	qRT-PCR analysis of *nigCII*
qRT-CIIR	TGGCGGGCATCAGCAGGGTG	qRT-PCR analysis of *nigCII*
qRT-AIF	AGGGCGGGAACGCCATCA	qRT-PCR analysis of *nigAI*
qRT-AIR	TCGACGCGGTCCAGGAAACC	qRT-PCR analysis of *nigAI*
qRT-EF	GGTGCTGGATGTCGGATGTGG	qRT-PCR analysis of *nigE*
qRT-ER	CGGATTCGGCAGCCTGTTTGA	qRT-PCR analysis of *nigE*
qRT-RF	TGGAATGCGTCGAGATGCTG	qRT-PCR analysis of *nigR*

qRT-RR	GGTGCAGTGCCTCCCGTAAA	qRT-PCR analysis of *nigR*
qRT-hrdBF	CTGACCAGATTCCGCCAACCC	qRT-PCR analysis of *hrdB*
qRT-hrdBR	GCCTCTGCGGCACTGACCAT	qRT-PCR analysis of *hrdB*
